# Utility of 2-thioxo-pyrido[2,3-*d*]pyrimidinone in synthesis of pyridopyrimido[2,1-*b*][1,3,5]-thiadiazinones and pyridopyrimido[2,1-*b*][1,3]thiazinones as antimicrobial agents

**DOI:** 10.1186/s13065-017-0286-0

**Published:** 2017-06-20

**Authors:** Yasser H. Zaki, Sobhi M. Gomha, Amany M. G. Mohamed

**Affiliations:** 1 Department of Chemistry, Faculty of Science, Beni-Suef University, Beni-Suef, 62514 Egypt; 2 Department of Chemistry, Faculty of Science and Humanity Studies at Al-Quwayiyah, Shaqra University, Al-Quwayiyah, 11971 Saudi Arabia; 3 Department of Chemistry, Faculty of Science, Cairo University, Giza, 12613 Egypt

**Keywords:** Pyridopyrimidinethione, Michael, Addition, Hydrazone, Bis-hydrazone, Pyrazolines, Antimicrobial agents

## Abstract

**Background:**

Pyridopyrimidines are of current interest because of their extensive variety of biological and pharmacological activities.

**Results:**

A series of pyrido[2′,3′:4,5]pyrimido[2,1-*b*][1,3,5]thiadiazinones was obtained by aminomethylation of pyridopyrimidinethione with formaldehyde solution (37%) and different primary aromatic amines. Another series of pyrido[2′,3:4,5]pyrimido[2,1-*b*][1,3]thiazinones was prepared by Michael addition reaction of pyridopyrimidinethione to the activated double bond of a number of arylidene malononitrile and 2-(benzo[*d*][1,3]dioxol-5-ylmethylene)malononitrile. The mechanisms of formation of the synthesized compounds were discussed and the assigned structure was established via microanalysis and spectral data (IR, ^1^H NMR, and Ms.). A comparative study of the biological activity of the synthesized compounds with chloramphenicol and trimethoprim/sulphamethoxazole is shown in Table 1. Generally, all synthesized compounds showed adequate inhibitory effects on the growth of Gram-positive and Gram-negative bacteria.

**Conclusions:**

In this study, we use a simple (synthetic) strategy for the synthesis of pyrimidothiadiazines, based on their aminomethylation through the Mannich reaction; they have also been synthesized by the application of the Michael addition to activated nitriles. Mechanisms and structures of the newly synthesized compounds were examined: the antimicrobial activity of some selected compounds was evaluated, which demonstrated adequate inhibitory effects.

**Graphical abstract:**
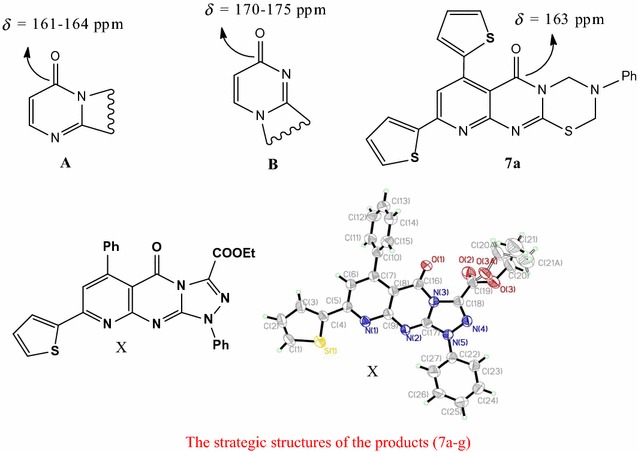
The strategic structures of the products (**7a**–**g**).

## Background

Pyridopyrimidines and their intertwined heterocyclic ring frameworks are of current interest [1–4]. Pyrido[2,3-*d*]pyrimidines are annulated uracil which, have gotten significant consideration over the last years because of their extensive variety of biological and pharmacological activities, such as anticancer [5–9], antimicrobial [10, 11], antiviral [12, 13], anti-inflammatory agents [14] antifolate [15, 16], PDE IV inhibitors [17], and Inhibitors for hepatitis B virus [18]. Also, pyridopyrimidine moiety was considered as the best-known tyrosine kinase inhibitor for the treatment of endless myelogenous leukemia and medication resistance rises by enhancement of the improvement of a transformation [19]. In the perspective of every one of these actualities mentioned above and as a major aspect of our program to hunt down, possibly bioactive new specialists [20–28], we report in this the union of novel pyridopyrimido[2,1-b][1,3,5]thiadizinone and pyridopyrimido[2,1-b][1,3]thiazinone derivatives. Moreover, the antimicrobial activities of the objective products were assessed.

## Results and discussion

### Chemistry

**Scheme 1:**
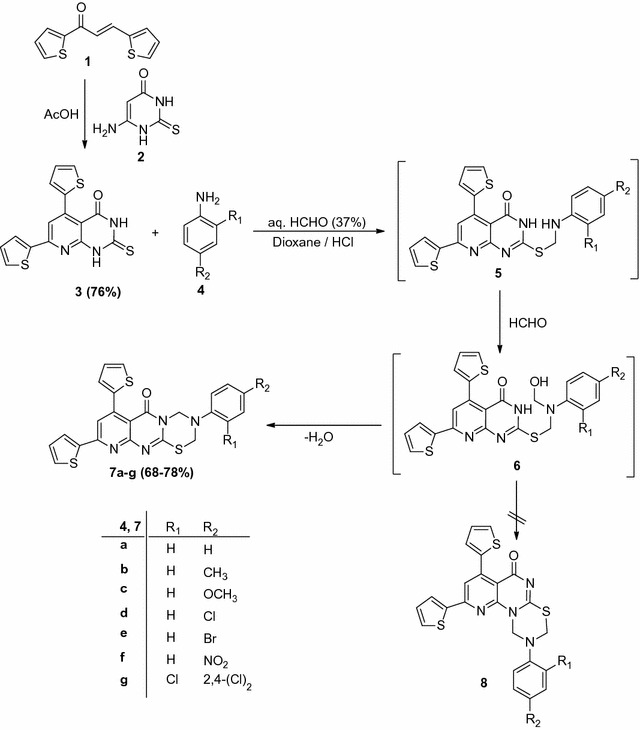
Synthesis of pyridopyrimidothiadiazinone derivatives (**7a**–**g**)

Treatment of 1,3-di(thiophen-2-yl)prop-2-en-1-one (**1**) [29] with 6-amino-2-thioxo-2,3-dihydropyrimidin-4(1*H*)-one (**2**) in presence of glacial acetic acid followed by acidifying with hydrochloric acid afforded 5,7-di(thiophen-2-yl)-2-thioxo-2,3-dihydropyrido[2,3-d]pyrimidin-4(1*H*)-one (**3**) (see Scheme 1). The reaction of 4,6-di(thiophen-2-yl)-3,4-dihydropyrimidine-2(1*H*)-thione (**3**) with each of the substituted anilines (**4a**–**g**) and excess aqueous formaldehyde solution (37%) in dioxane in the presence of a few drops of conc. hydrochloric acid afforded 3,7,9-triaryl-3,4-dihydropyrido[2′,3′:4,5]pyrimido[2,1-*b*][1,3,5]thiadiazin-6(2*H*)-ones (**7a**–**g**) as a single product as evidenced by TLC analysis of the crude product. The elemental analysis and mass spectral data of the isolated products were consistent with the compound (**7**) (see Scheme 1). The chemical structure of the compounds (**7a**–**g**) was confirmed based on elemental analysis and spectral information. The ^1^H NMR (DMSO-*d*_6_) spectrum of compound (**7a**) showed signals at *δ* = 4.83 (s, 2H, CH_2_), 5.43 (s, 2H, CH_2_), 6.57–7.84 (m, 11H, Ar–H), and 8.03 (s, 1H, pyridine-H5). It’s IR spectrum revealed absorption bands at 1597 cm^−1^ (C=N), 1648 cm^−1^ (C=O), 2923, 3063 cm^−1^ (C–H) (Scheme 1).

**Fig. 1:**
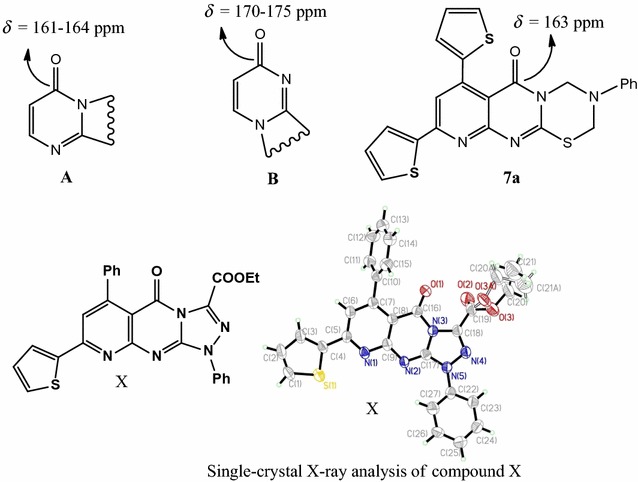
The strategic structures of the products (**7a**–**g**)

According to a survey of the literature [29–34], the S-alkylated pyrimidines cyclization occurs at N-atom, adjoining to the C=O group instead of the other N-molecule, based on ^13^C NMR data. Thus, the ^13^C NMR spectral data of compound (**7a**) shows carbonyl carbon signals of the pyrimidinone at 162 ppm, indicating that the N-atom adjoining to C=O is sp^3^-hybridized, which is different from C=O adjoining a sp^2^-hybridized nitrogen that usually appears at 170–175 ppm. [29]. Therefore, the structure of compound (**7b**) is found in one form namely, (**A**), rather than (**B**). Fares et al. recently confirmed that the cyclization carried out at N-atom, adjoining to the C=O group based on single-crystal X-ray analysis [35]; so, the structures of the products (**7a**–**g**) being formulated as linear isomers (**A**) rather than isomeric angular isomers (**B**) as represented in Fig. 1.

In light of the aforementioned results, the mechanism summarized in Scheme 1 represents the most appropriate pathway for the formation of (**7a**–**g**) from the reaction of thione (**3**) with the appropriate amines (**4a**–**g**), and formaldehyde solution. The reaction involves initial formation of intermediate compound (**5**), which undergoes addition of another formaldehyde molecule as soon as it is formed to give the S-alkylated pyrimidinones (**6**). The intermediate compound so formed (**6**) undergo in situ cyclization as soon as they are formed, via elimination of a water molecule to afford the targets compounds (**7a**–**g**) (Scheme 1).

**Scheme 2:**
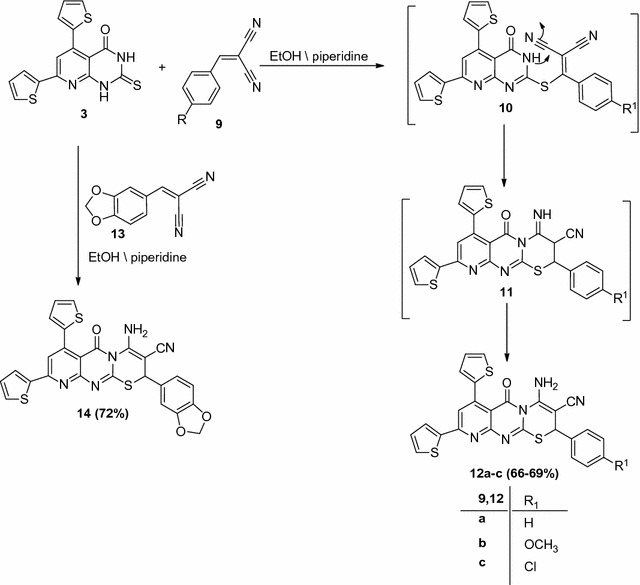
Synthesis of pyridopyrimidothiazinone derivatives (**12a**–**c**) and (**14**)

Another group of fused pyrimidothiazinones was designed by treatment of pyridopyrimidinethione (**3**) with each of the appropriate arylidene malononitrile (**9a**–**c**) in refluxing ethanol in the presence of a catalytic amount of piperidine afforded the pyridopyrimidothiazinones (**12a**–**c**) by application of Michael’s addition reaction. The structures of compounds (**12a**–**c**) were confirmed by elemental analysis and spectral data. In each case the IR spectra of (**12a**–**c**) revealed three absorption bands near ν = 1656, 2192, 3184 and 3427 cm^−1^ attributed to the carbonyl, nitrile and the amino groups. The ^1^H NMR spectrum of (**12a**) showed signals at δ = 4.80 (s, 1H, CH), 6.87–7.78 (m, 11H, Ar–H), 8.01 (s, 1H, pyridine-H5), and 9.30 (s, 2H, NH_2_, D_2_O exchangeable) (see “Experimental section”). The mass spectra of products (**12**) appeared in each case a molecular ion peak which was compatible with the molecular formula of the assigned structure. A plausible mechanism was summarized (see Scheme 2) to demonstrate the formation of products (**12**). It was proposed that the reaction of pyridopyrimidinethione (**3**) with arylidene malononitrile carried out by initial Michael’s addition reaction of the thiol group to the activated double bond of compound (**10**) to give the non-isolable intermediate (**11**), which undergo tandem intramolecular cyclization and tautomerism to afford the final products (**12**) (Scheme 2).

In the same way, treatment of 4,6-di(thiophen-2-yl)-3,4-dihydropyrimidine-2(1*H*)-thione (**3**) with 2-(benzo[d][1,3]dioxol-5-ylmethylene)malononitrile (**13**) afforded pyridopyrimidothiazinone derivative (**14**). The reaction takes place by the Michael’s addition reaction of thione (**3**) to (**13**) under the same reaction conditions (Scheme 2). The chemical structure of the compound (**14**) was confirmed based on elemental analysis, and spectral data. The ^1^H NMR (DMSO-*d*_6_) spectrum of compound (**14**) showed signals at *δ* = 4.62 (s, 1H, CH), 5.99 (s, 2H, CH_2_), 6.75–7.82 (m, 9H, Ar–H), 8.05 (s, 1H, pyridine-H5), and 9.81 (s, 2H, NH_2_, D_2_O exchangeable). It’s IR spectrum revealed absorption bands at 1592 cm^−1^ (C=N), 1685 cm^−1^ (C=O), 2190 cm^−1^ (CN), 2938, 3074 cm^−1^ (C–H), 3188, 3432 cm^−1^ (NH_2_) (Scheme 2).

**Scheme 3:**
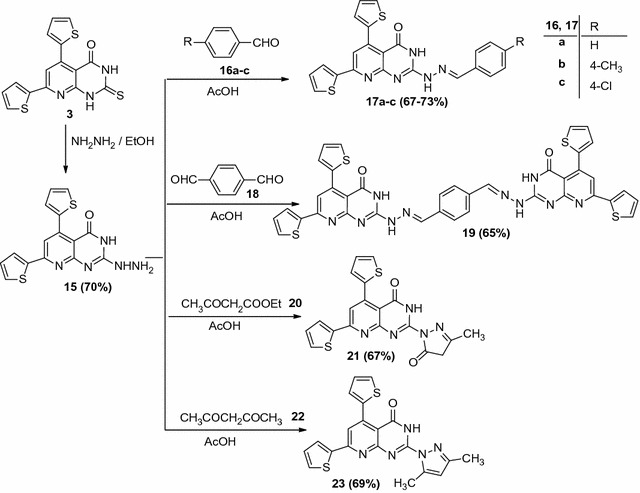
Synthesis of pyridopyrimidine derivatives (**17a**–**c**), (**19**), (**21**) and (**23**)

The compound 4,6-di(thiophen-2-yl)-3,4-dihydropyrimidine-2(1*H*)-thione (**3**) was reacted with hydrazine hydrate in refluxing ethanol to afford 2-hydrazinyl-5,7-di(thiophen-2-yl)pyrido[2,3-d]pyrimidin-4(3*H*)-one (**15**). The chemical structure of the compound (**15**) was confirmed based on elemental analysis, spectral data and chemical transformation. The ^1^H NMR (DMSO-*d*_6_) spectrum of compound (**15**) showed signals at *δ* = 2.88 (s, D_2_O exchangeable, 2H, NH_2_), 4.87 (s, D_2_O exchangeable, 1H, NH), 6.57–7.96 (m, 6H, Ar–H), 8.26 (s, 1H, pyridine-H5), and 9.23 (s, D_2_O exchangeable, 1H, NH). It’s IR spectrum revealed absorption bands at 1600 cm^−1^ (C=N), 1635 cm^−1^ (C=O), 2924, 3096 cm^−1^ (C–H), 3179–3423 cm^−1^ (NH_2_ and 2NH). Therefore, treatment of 2-hydrazinyl-5,7-di(thiophen-2-yl)pyrido[2,3-*d*]pyrimidin-4(3*H*)-one (**15**) with each of the appropriate aldehydes (**16a**–**c**) and terephthaldehyde (**18**) in refluxing acetic acid in the presence of a few drops of concentrated hydrochloric acid afforded the corresponding hydrazones derivatives (**17a**–**c**) and bis-hydrazone (**19**), respectively. The chemical structures of the compound (**17a**–**c**) and (**19**) (Scheme 3) were confirmed based on elemental analysis and spectral data. For example, the ^1^H NMR (DMSO-*d*_6_) spectrum of compound (**17a**) showed signals at *δ* = 7.11–7.96 (m, 11H, Ar–H), 8.01 (s, 1H, pyridine-H5), 8.10 (s, 1H, CH=N), and 11.39, 11.86 (2s, 2H, 2NH, D_2_O exchangeable). It’s IR spectrum revealed absorption bands at 1591 cm^−1^ (C=N), 1646 cm^−1^ (C=O), 2924, 3023 cm^−1^ (C–H), 3165, 3447 cm^−1^ (2NH).

Also, 2-hydrazinyl-5,7-di(thiophen-2-yl)pyrido[2,3-d]pyrimidin-4(3*H*)-one (**15**) reacted with ethyl acetoacetate (**20**) or acetyl acetone (**22**) in refluxing acetic acid to give pyrazolines (**21**) and (**23**), respectively. The chemical structures of the compounds (**21**) and (**23**) were confirmed based on elemental analysis and spectral data. For example, the ^1^H NMR (DMSO-*d*_6_) spectrum of compound (**23**) showed signals at *δ* = 1.89 (s, 3H, CH_3_), 2.24 (s, 3H, CH_3_), 6.17 (s, 1H, pyrazole-H4), 6.90–7.82 (m, 6H, Ar–H), 8.03 (s, 1H, pyridine-H5), and 11.20 (s, 1H, NH, D_2_O exchangeable). It’s IR spectrum revealed absorption bands at 1601 cm^−1^ (C=N), 1634 cm^−1^ (C=O), 2924, 3096 cm^−1^ (C–H), 3343 cm^−1^ (NH).

### Biological screening

#### Antimicrobial activity

In-vitro antimicrobial screening of compounds **7a**–**g**, **12a**–**c**, **14**, **17a**–**c**, **19**, **21** and **23** prepared for the study were carried out using cultures of two fungal strains, namely, *Candida albicans* (ATCC 10231) (CA) and *Aspergillus niger* (ATCC) (AN), as well as four bacterial species, namely, Gram-positive bacteria, *Staphylococcus aureus* (ATCC 29213) (SA), and *Bacillus subtilus* (ATCC 6051) (BS), Gram-negative bacteria, and *Escherichia coli* (ATCC 25922) (EC). *Chloramphenicol* and *Miconazole* are used as antibacterial and antifungal reference drugs to evaluate the potency of the tested compounds under the same conditions (Table 1).

**Table 1:** Antimicrobial activity expressed as inhibition diameter zones in a centimeter (cm) of tested compounds against the pathogenically stains based on disk diffusion as the assay

Compound no.	Fungi		G^+^ bacteria		G^−^ bacteria
	AN	CA	SA	BS	EC
**7a**	NA	20	17	18	18
**7b**	NA	NA	18	20	15
**7c**	NA	8	21	18	12
**7d**	NA	8	18	14	13
**7e**	NA	NA	22	12	17
**7f**	NA	24	18	15	23
**7g**	NA	22	25	21	13
**12a**	NA	25	19	17	11
**12b**	NA	25	18	14	19
**12c**	NA	20	15	13	25
**14**	NA	21	20	16	19
**17a**	NA	11	15	14	16
**17b**	NA	13	14	21	20
**17c**	NA	14	16	9	11
**19**	NA	22	25	15	8
**21**	NA	11	12	9	10
**23**	NA	11	10	13	13
Chloramphenicol	–	–	30	24	29
Miconazole	28	26	–	–	–
DMSO	NA	NA	NA	NA	NA

### Conclusions

In this study, we use a simple (synthetic) strategy for the synthesis of pyrimidothiadiazines, based on their aminomethylation through the Mannich reaction; they have also been synthesized by the application of the Michael addition to activated nitriles. Mechanisms and structures of the newly synthesized compounds were examined: the antimicrobial activity of some selected compounds was evaluated, which demonstrated adequate inhibitory effects.

## Experimental section

### General methods

Melting points were recorded on a Gallenkamp electrothermal apparatus, with infrared spectra (KBr) determined on a Pye Unicam SP-3000 (Cambridge, UK) infrared spectrophotometer. ^1^H NMR was assessed on a Varian Gemini 300 spectrometer (300 MHz) (Raleigh, NC, USA) in DMSO-d_6_ with TMS as an internal standard. Mass spectra were recorded on a GCMS-QP 1000 EX Shimadzu spectrometer. We conduct elemental analyses at the Microanalytical Center, University of Cairo, Giza, Egypt.

#### Preparation of 5,7-di(thiophen-2-yl)-2-thioxo-2,3-dihydropyrido[2,3-*d*]pyrimidin-4(1*H*)-one (**3**)

A mixture of chalcone (**1**) (2.20 g, 10 mmol) and 6-amino-2-thioxo-2,3,4-trihydro-1*H*-pyrimidin-4-one (**2**) (1.43 g, 10 mmol) in glacial acetic acid (30 mL) was heated under reflux for 6 h after cooling, the reaction mixture was then poured into an ice/HCl mixture, and the solid product was collected and recrystallized from acetic acid as yellow solid, yield 76%, mp 236–238 °C; IR (KBr, cm^−1^) ν = 3426, 3281 (2NH), 3038, 2916 (C–H), 1643 (C=O), 1602 (C=N); ^1^H NMR (DMSO-*d*_6_) at *δ* = 6.67–8.33 (m, 6H, Ar–H), 8.37 (s, 1H, pyrimidine-H), 11.43, 12.11 (2s, 2H, 2NH, exchangeable with D_2_O); MS *m*/*z* (%) 343 (M^+^, 100), 310 (19), 228 (18), 171 (23), 111 (17), 40 (25). Calculated combustion elemental analysis (Anal. Calcd.) for C_15_H_9_N_3_OS_3_ (342.99): C, 52.46; H, 2.64; N, 12.23. Found: C, 52.33; H, 2.51; N, 12.04%.

#### Synthesis of 3,7,9-triaryl-3,4-dihydropyrido[2′,3′:4,5]pyrimido[2,1-*b*][1,3,5]-thiadiazin-6(2*H*)-ones (**7a**–**g**)

*General procedure* A mixture of thione (**3**) (0.343 g, 1 mmol), 37% formaldehyde solution (2 mL) and the appropriate aniline derivative (**4a**–**g**) (1 mmol) in dioxane (20 mL) in the presence of few drops of HCl was stirred at room temperature for 4–8 h (monitored by TLC). The solid that precipitated was filtered off, washed with water, dried and finally crystallized from dioxane or EtOH to give the respective products (**7a**–**g**). The physical and spectral data of products (**7a**–**g**) are depicted as follows.

##### 3-Phenyl-7,9-di(thiophen-2-yl)-3,4-dihydropyrido[2′,3′:4,5]pyrimido[2,1-*b*][1,3,5]thiadiazin-6(2*H*)-one (**7a**)

Yellow solid; yield 72%; mp 186–190 °C (dioxane); IR (KBr): *v*_max_ = 1597 (C=N), 1648 (C=O), 2923, 3063 (C–H) cm^−1^; ^1^H NMR (300 MHz, DMSO-*d*_*6*_): *δ* = 4.83 (s, 2H, CH_2_), 5.43 (s, 2H, CH_2_), 6.57–7.84 (m, 11H, Ar–H), 8.03 (s, 1H, pyridine-H5); MS (70 eV): *m*/*z* = 460 (M^+^, 12), 377 (55), 253 (82), 170 (69), 64 (100). Calculated combustion elemental analysis (Anal. Calcd.) for C_23_H_16_N_4_OS_3_ (460.05): C, 59.98; H, 3.50; N, 12.16. Found: C, 59.90; H, 3.34; N, 12.03%.

##### 7,9-Di(thiophen-2-yl)-3-(*p*-tolyl)-3,4-dihydropyrido[2′,3′:4,5]pyrimido[2,1-*b*][1,3,5]thiadiazin-6(2*H*)-one (**7b**)

Yellow solid; yield 75%; mp 153–155 °C (dioxane); IR (KBr): *v*_max_ = 1596 (C=N),1644 (C=O), 2920, 3029 (C–H) cm^−1^; ^1^H NMR (300 MHz, DMSO-*d*_*6*_): *δ* = 2.23 (s, 3H, CH_3_), 5.42 (s, 2H, CH_2_), 5.79 (s, 2H, CH_2_), 6.66–7.99 (m, 10H, Ar–H), 8.11 (s, 1H, pyridine-H5); MS (70 eV): *m*/*z* = 474 (M^+^, 8), 342 (48), 221 (70), 77 (81), 59 (96), 40 (100). Calculated combustion elemental analysis (Anal. Calcd.) for C_24_H_18_N_4_OS_3_ (474.06): C, 60.73; H, 3.82; N, 11.80. Found: C, 60.79; H, 3.69; N, 11.66%.

##### 3-(4-Methoxyphenyl)-7,9-di(thiophen-2-yl)-3,4-dihydropyrido[2′,3′:4,5]pyrimido-[2,1-*b*][1,3,5]-thiadiazin-6(2*H*)-one (**7c**)

Pale green solid; yield 68%; mp 131–133 °C (EtOH); IR (KBr): *v*_max_ = 1592 (C=N),1638 (C=O), 2923, 3043 (C–H) cm^−1^; ^1^H NMR (300 MHz, DMSO-*d*_*6*_): *δ* = 3.67 (s, 3H, OCH_3_), 5.38 (s, 2H, CH_2_), 5.74 (s, 2H, CH_2_), 6.64–7.83 (m, 10H, Ar–H), 8.00 (s, 1H, pyridine-H5); MS (70 eV): *m*/*z* = 490 (M^+^, 9), 469 (52), 342 (42), 171 (38), 111 (63), 44 (100). Calculated combustion elemental analysis (Anal. Calcd.) for C_24_H_18_N_4_O_2_S_3_ (490.06): C, 58.75; H, 3.70; N, 11.42. Found: C, 58.53; H, 3.51; N, 11.58%.

##### 3-(4-Chlorophenyl)-7,9-di(thiophen-2-yl)-3,4-dihydropyrido[2′,3′:4,5]pyrimido[2,1-*b*][1,3,5]-thiadiazin-6(2*H*)-one (**7d**)

Brown solid; yield 78%; mp 192–194 °C (dioxane); IR (KBr): *v*_max_ = 1592 (C=N),1646 (C=O), 2920, 2957, 3099 (C–H) cm^−1^; ^1^H NMR (300 MHz, DMSO-*d*_*6*_): *δ* = 5.42 (s, 2H, CH_2_), 5.79 (s, 2H, CH_2_), 6.61–7.92 (m, 10H, Ar–H), 8.03 (s, 1H, pyridine-H5); MS (70 eV): *m*/*z* = 496 (M^+^+2, 3), 494 (M^+^, 10), 327 (52), 192 (48), 131 (100), 62 (70). Calculated combustion elemental analysis (Anal. Calcd.) for C_23_H_15_ClN_4_OS_3_ (494.01): C, 55.80; H, 3.05; N, 11.32. Found: C, 55.89; H, 3.02; N, 11.17%.

##### 3-(4-Bromophenyl)-7,9-di(thiophen-2-yl)-3,4-dihydropyrido[2′,3′:4,5]pyrimido[2,1-*b*][1,3,5]-thiadiazin-6(2*H*)-one (**7e**)

Yellow solid; yield 77%; mp 173–175 °C (dioxane); IR (KBr): *v*_max_ = 1590 (C=N),1650 (C=O), 2912, 2956, 3095 (C–H) cm^−1^; ^1^H NMR (300 MHz, DMSO-*d*_*6*_): *δ* = 5.40 (s, 2H, CH_2_), 5.81 (s, 2H, CH_2_), 6.84–7.92 (m, 10H, Ar–H), 8.04 (s, 1H, pyridine-H5); MS (70 eV): *m*/*z* = 540 (M^+^+2, 7), 538 (M^+^, 8), 343 (53), 228 (50), 111 (100), 45 (95). Calculated combustion elemental analysis (Anal. Calcd.) for C_23_H_15_BrN_4_OS_3_ (537.96): C, 51.21; H, 2.80; N, 10.39. Found: C, 51.37; H, 2.64; N, 10.18%.

##### 3-(4-Nitrophenyl)-7,9-di(thiophen-2-yl)-3,4-dihydropyrido[2′,3′:4,5]pyrimido[2,1-*b*][1,3,5]-thiadiazin-6(2*H*)-one (**7f**)

Brown solid; yield 75%; mp 160–162 °C (dioxane); IR (KBr): *v*_max_ = 1596 (C=N),1654 (C=O), 2911, 2957, 3094 (C–H) cm^−1^; ^1^H NMR (300 MHz, DMSO-*d*_*6*_): *δ* = 5.40 (s, 2H, CH_2_), 5.74 (s, 2H, CH_2_), 6.67–7.97 (m, 10H, Ar–H), 8.03 (s, 1H, pyridine-H5); MS (70 eV): *m*/*z* = 505 (M^+^, 14), 344 (37), 191 (51), 111 (86), 57 (100), 43 (84). Calculated combustion elemental analysis (Anal. Calcd.) for C_23_H_15_N_5_O_3_S_3_ (505.03): C, 54.64; H, 2.99; N, 13.85. Found: C, 54.48; H, 2.91; N, 13.69%.

##### 3-(2,4-Dichlorophenyl)-7,9-di(thiophen-2-yl)-3,4-dihydropyrido[2′,3′:4,5]pyrimido-[2,1-*b*][1,3,5]-thiadiazin-6(2*H*)-one (**7g**)

Yellow solid; yield 77%; mp 135–137 °C (DMF); IR (KBr): *v*_max_ = 1593 (C=N),1649 (C=O), 2911, 2959, 3095 (C–H) cm^−1^; ^1^H NMR (300 MHz, DMSO-*d*_*6*_): *δ* = 5.32 (s, 2H, CH_2_), 5.78 (s, 2H, CH_2_), 6.75–7.42 (m, 8H, Ar–H), 7.74 (s, 1H, Ar–H), 8.10 (s, 1H, pyridine-H5); MS (70 eV): *m*/*z* = 527 (M^+^, 8), 446 (27), 220 (100), 187 (52), 82 (89), 43 (58). Calculated combustion elemental analysis (Anal. Calcd.) for C_23_H_14_Cl_2_N_4_OS_3_ (527.97): C, C, 52.17; H, 2.67; N, 10.58. Found: C, 52.29; H, 2.48; N, 10.49%.

#### Synthesis of pyridopyrimidothiazinone derivatives (**12a**–**c**) and (**14**)

*General procedure* To a solution of thione (**3**) (0.343 g, 1 mmol), an appropriate amount of arylidenemalononitrile (**7a**–**c**), and (**13**) (1 mmol) 20 mL of ethanol (EtOH), 0.5 mL of piperidine was added. The mixture so obtained was refluxed for 8 h. The solid substance that precipitated after cooling was filtered off, washed with water, dried and finally crystallized from EtOH to give products (**12a**–**c**) and (**14**), respectively.

##### 4-Amino-6-oxo-2-phenyl-7,9-di(thiophen-2-yl)-2,6-dihydropyrido[2′,3′:4,5]pyrimido-[2,1-*b*][1,3]-thiazine-3-carbonitrile (**12a**)

Brown solid; yield 68%; mp 163–165 °C; IR (KBr): *v*_max_ = 1591 (C=N),1626 (C=O), 2185 (CN), 2937, 3067 (C–H), 3193, 3413 (NH_2_) cm^−1^; ^1^H NMR (300 MHz, DMSO-*d*_*6*_): *δ* = 4.80 (s, 1H, CH), 6.87–7.78 (m, 11H, Ar–H), 8.01 (s, 1H, pyridine-H5), 9.30 (s, 2H, NH_2_, D_2_O exchangeable); MS (70 eV): *m*/*z* = 497 (M^+^, 17), 314 (38), 211 (71), 172 (52), 77 (82), 43 (100). Calculated combustion elemental analysis (Anal. Calcd.) for C_25_H_15_N_5_OS_3_ (497.04): C, 60.34; H, 3.04; N, 14.07. Found: C, 60.39; H, 3.15; N, 13.94%.

##### 4-Amino-2-(4-methoxyphenyl)-6-oxo-7,9-di(thiophen-2-yl)-2,6-dihydropyrido-[2′,3′:4,5]pyrimido-[2,1-*b*][1,3]thiazine-3-carbonitrile (**12b**)

Brown solid; yield 66%; mp 167–169 °C; IR (KBr): *v*_max_ = 1590 (C=N),1630 (C=O), 2200 (CN), 2930, 3052 (C–H), 3193, 3427 (NH_2_) cm^−1^; ^1^H NMR (300 MHz, DMSO-*d*_*6*_): *δ* = 3.81 (s, 3H, OCH_3_), 4.63 (s, 1H, CH), 6.83–7.75 (m, 10H, Ar–H), 7.98 (s, 1H, pyridine-H5), 9.43 (s, 2H, NH_2_, D_2_O exchangeable); MS (70 eV): *m*/*z* = 527 (M^+^, 6), 305 (36), 211 (39), 153 (64), 80 (93), 64 (100). Calculated combustion elemental analysis (Anal. Calcd.) for C_26_H_17_N_5_O_2_S_3_ (527.05): C, 59.18; H, 3.25; N, 13.27. Found: C, 59.04; H, 3.16; N, 13.03%.

##### 4-Amino-2-(4-chlorophenyl)-6-oxo-7,9-di(thiophen-2-yl)-2,6-dihydropyrido[2′,3′:4,5]pyrimido[2,1-*b*][1,3]thiazine-3-carbonitrile (**12c**)

Brown solid; yield 69%; mp 204–206 °C; IR (KBr): *v*_max_ = 1593 (C=N),1684 (C=O), 2194 (CN), 2942, 3073 (C–H), 3165, 3437 (NH_2_) cm^−1^; ^1^H NMR (300 MHz, DMSO-*d*_*6*_): *δ* = 4.84 (s, 1H, CH), 6.85–7.83 (m, 10H, Ar–H), 8.06 (s, 1H, pyridine-H5), 9.79 (s, 2H, NH_2_, D_2_O exchangeable); MS (70 eV): *m*/*z* = 533 (M^+^+2, 1), 531 (M^+^, 4), 330 (64), 211 (54), 158 (66), 80 (53), 64 (100). Calculated combustion elemental analysis (Anal. Calcd.) for C_25_H_14_ClN_5_OS_3_ (531.00): C, 56.43; H, 2.65; N, 13.16. Found: C, 56.62; H, 2.63; N, 13.10%.

##### 4-Amino-2-(benzo[*d*][1,3]dioxol-5-yl)-6-oxo-7,9-di(thiophen-2-yl)-2,6-dihydropyrido[2′,3′:4,5]pyrimido[2,1-*b*][1,3]thiazine-3-carbonitrile (**14**)

Brown solid; yield 72%; mp 213–215 °C; IR (KBr): *v*_max_ = 1592 (C=N), 1685 (C=O), 2190 (CN), 2938, 3074 (C–H), 3188, 3432 (NH_2_) cm^−1^; ^1^H NMR (300 MHz, DMSO-*d*_*6*_): *δ* = 4.62 (s, 1H, CH), 5.99 (s, 2H, CH_2_), 6.75–7.82 (m, 9H, Ar–H), 8.05 (s, 1H, pyridine-H5), 9.81 (s, 2H, NH_2_, D_2_O exchangeable); MS (70 eV): *m*/*z* = 541 (M^+^, 12), 402 (51), 309 (63), 211 (64), 80 (100), 57 (84). Calculated combustion elemental analysis (Anal. Calcd.) for C_26_H_15_N_5_O_3_S_3_ (541.62): C, 57.66; H, 2.79; N, 12.93. Found: C, 57.42; H, 2.70; N, 12.62%.

#### Synthesis of 2-hydrazinyl-5,7-di(thiophen-2-yl)pyrido[2,3-*d*]pyrimidin-4(3*H*)-one (**15**)

Hydrazine hydrate (80%, 20 mL) was added to thione (**3**) (3.43 g, 10 mmol) in the presence of dry EtOH (40 mL), and the reaction mixture was kept under reflux for 30 h and then cooled. The precipitated solid was filtered off and crystallized from dimethylformamide (DMF) to give (**4**) as a white solid, mp 325–327 °C; 70% yield; IR (KBr): *v*_max_ = 1600 (C=N), 1635 (C=O), 2924, 3096 (C–H), 3179–3423 (NH_2_ and 2NH), cm^−1^; ^1^H NMR (300 MHz, DMSO-*d*_*6*_): *δ* = 2.88 (s, D_2_O exchangeable, 2H, NH_2_), 4.87 (s, D_2_O exchangeable, 1H, NH), 6.57–7.96 (m, 6H, Ar–H), 8.26 (s, 1H, pyridine-H5), 9.23 (s, D_2_O exchangeable, 1H, NH); MS (70 eV): *m*/*z* = 341 (M^+^, 28), 232 (64), 203 (47), 111 (100), 97 (54), 58 (68). Calculated combustion elemental analysis (Anal. Calcd.) for C_15_H_11_N_5_OS_2_ (341.04): C, 52.77; H, 3.25; N, 20.51%. Found: C, 52.64; H, 3.14; N, 20.35%.

#### Synthesis of hydrazones (**17a**–**c**)

A mixture of hydrazine derivative (**15**) (0.341 g, 1 mmol) and an appropriate amount of aldehyde (**16a**–**c**) (1 mmol) in acetic acid (20 mL), and a few drops of concentrated hydrochloric acid (≈1 mL) were heated under reflux for 5 h. The resultant mixture obtained was then cooled and diluted with water. The formed solid product was then collected by filtration, dried and recrystallized from DMF to afford the corresponding hydrazones (**17a**–**c**).

##### 2-(2-Benzylidenehydrazinyl)-5,7-di(thiophen-2-yl)pyrido[2,3-d]pyrimidin-4(3*H*)-one (**17a**)

Yellow solid; yield 73%; mp 187–189 °C; IR (KBr): *v*_max_ = 1591 (C=N),1646 (C=O), 2924, 3023 (C–H), 3165, 3447 (2NH) cm^−1^; ^1^H NMR (300 MHz, DMSO-*d*_*6*_): *δ* = 7.11–7.96 (m, 11H, Ar–H), 8.01 (s, 1H, pyridine-H5), 8.10 (s, 1H, CH=N), 11.39, 11.86 (2s, 2H, 2NH, D_2_O exchangeable); MS (70 eV): *m*/*z* = 429 (M^+^, 85), 352 (100), 310 (41), 171 (38), 90 (29). Calculated combustion elemental analysis (Anal. Calcd.) for C_22_H_15_N_5_OS_2_ (429.07): C, 61.52; H, 3.52; N, 16.31. Found: C, 61.59; H, 3.37; N, 16.18%.

##### 2-(2-(4-Methylbenzylidene)hydrazinyl)-5,7-di(thiophen-2-yl)pyrido[2,3-*d*]pyrimidin-4(3*H*)-one (**17b**)

Yellow solid; yield 69%; mp 181–183 °C; IR (KBr): *v*_max_ = 1591 (C=N),1685 (C=O), 2922, 3090 (C–H), 3169, 3416 (2NH) cm^−1^; ^1^H NMR (300 MHz, DMSO-*d*_*6*_): *δ* = 2.38 (s, 3H, CH_3_), 7.09–8.05 (m, 10H, Ar–H), 8.19 (s, 1H, pyridine-H5), 8.63 (s, 1H, CH=N), 11.39, 11.90 (2s, 2H, 2NH, D_2_O exchangeable); MS (70 eV): *m*/*z* = 443 (M^+^, 9), 352 (63), 220 (39), 117 (37), 91 (72), 64 (100). Calculated combustion elemental analysis (Anal. Calcd.) for C_23_H_17_N_5_OS_2_ (443.09): C, 62.28; H, 3.86; N, 15.79. Found: C, 62.35; H, 3.64; N, 15.58%.

##### 2-(2-(4-Chlorobenzylidene)hydrazinyl)-5,7-di(thiophen-2-yl)pyrido[2,3-d]pyrimidin-4(3*H*)-one (**17c**)

Yellow solid; yield 67%; mp 230–232 °C; IR (KBr): *v*_max_ = 1590 (C=N),1683 (C=O), 2924, 3074 (C–H), 3193, 3417 (2NH) cm^−1^; ^1^H NMR (300 MHz, DMSO-*d*_*6*_): *δ* = 7.11–8.02 (m, 10H, Ar–H), 8.08 (s, 1H, pyridine-H5), 8.69 (s, 1H, CH=N), 11.53, 11.90 (2s, 2H, 2NH, D_2_O exchangeable); MS (70 eV): *m*/*z* = 465 (M^+^+2, 5), 463 (M^+^, 12), 352 (37), 137 (83), 64 (100). Calculated combustion elemental analysis (Anal. Calcd.) for C_22_H_14_ClN_5_OS_2_ (463.03): C, 56.95; H, 3.04; N, 15.09. Found: C, 56.77; H, 3.08; N, 14.86%.

#### Synthesis of bis-hydrazone (**19**)

A mixture of hydrazine derivative (**15**) (0.682 g, 2 mmol) and terephthaldehyde (**18**) (0.134 g, 1 mmol) in acetic acid (20 mL) and a few drops of concentrated hydrochloric acid (≈1 mL) were heated under reflux for 6 h. The reaction mixture was then cooled and diluted with water. The formed solid product was then collected by filtration, dried and recrystallized from DMF to obtain bis-hydrazone (**19**) as a brown residue with 65% yield. mp 307–309 °C; IR (KBr): *v*_max_ = 1594 (C=N), 1675 (C=O), 2925, 3023 (C–H), 3188, 3433 (2NH) cm^−1^; ^1^H NMR (300 MHz, DMSO-*d*_*6*_): *δ* = 6.96–8.09 (m, 16H, Ar–H), 8.14 (s, 2H, 2pyridine-H5), 8.70 (s, 2H, 2CH=N), 11.47 (s, 2H, 2NH, D_2_O exchangeable), 11.82 (s, 2H, 2NH, D_2_O exchangeable); MS (70 eV): *m*/*z* = 780 (M^+^, 16), 480 (35), 362 (40), 130 (19), 64 (100). Calculated combustion elemental analysis (Anal. Calcd.) for C_38_H_24_N_10_O_2_S_4_ (780.10): C, 58.44; H, 3.10; N, 17.94. Found: C, 58.59; H, 3.03; N, 17.75%.

#### Synthesis of pyrazolines (**21**) and (**23**)

A mixture of hydrazine derivative (**15**) (0.341 g, 1 mmol) and ethyl acetoacetate (**20**) or acetylacetone (**22**) (1 mmol) in acetic acid (20 mL) were heated under reflux for 5 h. The product started to separate out during the course of the reaction. The solid product was filtered, washed with water, dried and recrystallized from ethanol to give the corresponding pyrazoline derivatives (**21**) and (**23**).

##### 2-(3-Methyl-5-oxo-4,5-dihydro-1*H*-pyrazol-1-yl)-5,7-di(thiophen-2-yl)pyrido[2,3-*d*]pyrimidin-4(3*H*)-one (**21**)

Brown solid; yield 67%; mp 188–190 °C; IR (KBr): *v*_max_ = 1601 (C=N),1631, 1694 (2C=O), 2920, 2964, 3099 (C–H), 3173 (NH) cm^−1^; ^1^H NMR (300 MHz, DMSO-*d*_*6*_): *δ* = 1.90 (s, 3H, CH_3_), 2.21 (s, 2H, CH_2_), 6.87–7.98 (m, 6H, Ar–H), 8.05 (s, 1H, pyridine-H5),11.21 (s, 1H, NH, D_2_O exchangeable); MS (70 eV): *m*/*z* = 407 (M^+^, 8), 319 (63), 230 (41), 179 (83), 64 (100). Calculated combustion elemental analysis (Anal. Calcd.) for C_19_H_13_N_5_O_2_S_2_ (407.05): C, 56.01; H, 3.22; N, 17.19. Found: C, 55.90; H, 3.42; N, 17.05%.

##### 2-(3,5-Dimethyl-1*H*-pyrazol-1-yl)-5,7-di(thiophen-2-yl)pyrido[2,3-d]pyrimidin-4(3*H*)-one (**23**)

Brown solid; yield 69%; mp 212–214 °C; IR (KBr): *v*_max_ = 1601 (C=N),1634 (C=O), 2924, 3096 (C–H), 3343 (NH) cm^−1^; ^1^H NMR (300 MHz, DMSO-*d*_*6*_): *δ* = 1.89 (s, 3H, CH_3_), 2.24 (s, 3H, CH_3_), 6.17 (s, 1H, pyrazole-H4), 6.90–7.82 (m, 6H, Ar–H), 8.03 (s, 1H, pyridine-H5),11.20 (s, 1H, NH, D_2_O exchangeable); MS (70 eV): *m*/*z* = 405 (M^+^, 15), 318 (42), 210 (59), 111 (100), 64 (92). Calculated combustion elemental analysis (Anal. Calcd.) for C_20_H_15_N_5_OS_2_ (405.07): C, 59.24; H, 3.73; N, 17.27. Found: C, 59.33; H, 3.57; N, 17.05%.

### Antimicrobial activity

Antimicrobial activity was determined using the agar disc diffusion assay method as described by Hossain et al. [36]. The tested organisms were sub-cultured on Trypticase soya agar medium (Oxoid Laboratories, UK) for bacteria and Sabouraud dextrose agar (Oxoid Laboratories, UK) for fungi. Chloramphenicol and Trimethoprim/sulphamethoxazole were used as a positive control and DMSO solvent as a negative control. The plates were done in duplicate and average zone of inhibition was calculated. Bacterial cultures were incubated at 37 °C for 24 h while the other fungal cultures were incubated at (25–30 °C) for 3–5 days. Antimicrobial activity was determined by measurement zone of inhibition.

### Media used

*Sabouraud dextrose agar* the medium used for isolation of pathogenic yeasts has the following composition (g L^−1^): glucose, 20; peptone, 10; agar, 25 and distilled water, 1 L, pH was adjusted at 5.4. The medium was autoclaved at 121 °C for 15 min.

*Trypticase soya agar (TSA)* the medium was used to cultivate tested bacteria. It contains (g L^−1^) Tryptone (Pancreatic Digest of Casein) 15.0 g, Soytone (Papaic Digest of Soybean Meal) 5.0 g, Sodium Chloride 5.0 g, Agar 15.0 g and distilled water 1 L. The medium was autoclaved at 121 °C for 15 min.

## Abbreviations

TSA: trypticase soya agar
(ATCC 10231) (CA): *Candida albicans*
(ATCC) (ASP): *Aspergillus niger*
(ATCC 29213) (SA): *Staphylococcus aureus*
(ATCC 6051) (BS): *Bacillus subtilis*
(ATCC 700603) (KP): *Klebsiella pneumoniae*
(ATCC 25922) (EC): *Escherichia coli*
MW: molecular weight
TLC: thin layer chromatography
